# MicroRNA-363-3p, negatively regulated by long non-coding RNA small nucleolar RNA host gene 5, inhibits tumor progression by targeting Aurora kinase A in colorectal cancer

**DOI:** 10.1080/21655979.2021.2018972

**Published:** 2022-02-15

**Authors:** Qiuyun Guo, Lujia Dong, Chenxiao Zhang, Dechun Liu, Ping Peng

**Affiliations:** a Department of Oncology, Tongji Hospital, Tongji Medical College, Huazhong University of Science and Technology, Wuhan Hubei, China; b Department of Gastrointestinal Surgery, Xi’an No. 3 Hospital, the Affiliated Hospital of Northwest University, Xi’an, Shaanxi Province, China

**Keywords:** Colorectal cancer, miR-363-3p, AURKA, SNHG5, proliferation, invasion and apoptosis

## Abstract

MicroRNA-363-3p (miR-363-3p), reportedly, exhibits a tumor-suppressive role in human malignancies. Herein, our research was designed to further explain the functions and molecular mechanisms of miR-363-3p in the progression of colorectal cancer (CRC). With in vitro models, this study found that miR-363-3p was markedly under-expressed in CRC tissues and cells, and its overexpression suppressed the viability, migration, and invasion of CRC cells, and promoted cell apoptosis, whereas inhibiting miR-363-3p expression exhibited an opposite role. Additionally, aurora kinase A (AURKA), capable of counteracting the impacts of miR-363-3p on malignant biological behaviors of CRC cells, was identified as a direct target of miR-363-3p. Besides, miR-363-3p was sponged by long non-coding RNA small nucleolar RNA host gene 5 (SNHG5), which suppressed miR-363-3p expression. This research shows that SNHG5/miR-363-3p/AURKA axis partakes in CRC progression.

## Introduction

Colorectal cancer (CRC) is known as the third most common cancer, specifically, with approximately 1.8 million newly diagnosed cases and 881,000 reported deaths worldwide in 2018 [1,2]. Despite diagnostic and therapeutic approaches developing at full speed, the 5-year disease-free survival rate of CRC is merely about 50% [3,4]. It is necessary to decipher the molecular mechanism of CRC tumorigenesis and develop new therapeutic targets for CRC treatment.

MicroRNA (miRNA), a small non-coding RNA transcript with 21–23 *nt* in length, represses the translation of target messenger RNA (mRNA) by binding to 3’- untranslated region (3’-UTR) [5]. It is a crucial participant involved in a series of biological processes [6,7]. Its role in cancer biology has been widely reported. For instance, miR-548b suppresses the proliferation of CRC cells via repressing WNT2 expression [8]. MiR-330 inhibits CRC progression by targeting BACH1 [9]. Additionally, microRNA-363-3p (miR-363-3p), whose expression is reportedly to be decreased in osteosarcoma, non-small cell lung cancer, and CRC, can regulate the viability, metastasis, and drug resistance of cancer cells [10–12]. Nonetheless, the specific role and underlying mechanism regarding miR-363-3p in CRC progression warrant further investigation.

Aurora kinase is recognized as a serine/threonine kinase involved in mitosis, including Aurora-A (AURKA), Aurora-B, and Aurora-C, and mainly regulates the functions of centrosome and microtubules, ensuring the correct separation of centrosome and the complete division of cytoplasm, and its gene mutation or overexpression is associated with tumorigenesis [13]. AURKA, abnormally expressed in multiple cancers, is closely related to the occurrence and development of tumors, such as lung cancer [14], head and neck squamous cell carcinoma [15], and CRC [16], etc. AURKA regulates Wnt and Ras-MAPK signaling pathways, thereupon promoting CRC progression [17]. Nevertheless, little is known concerning the exact function and underlying mechanism of AURKA in CRC.

Long non-coding RNA (lncRNA), an RNA transcript with more than 200 *nt* in length, which is capable of interacting with RNA, DNA, or protein, regulates a lot of biological processes [18,19]. Reportedly, the dysregulation of lncRNA expression contributes to tumorigenesis and cancer progression [20–22]. LncRNA small nucleolar RNA host gene 5 (SNHG5), which is initially reported in B cell lymphoma, is oncogenic in several cancers [23–26]. Besides, SNHG5 shows high expression in CRC, and knocking down SNHG5 can suppress the viability, migration, and invasion of CRC cells [26]. SNHG5 sponges multiple miRNAs to participate in tumorigenesis [26–29]. Specifically, SNHG5 promotes the progression of renal cell carcinoma and liver cancer by acting as a molecular sponge for miR-363-3p [28,29]. However, to date, the regulatory relationship between SNHG5 and miR-363-3p in CRC has not yet been elucidated.

Based on the findings of previous studies, we hypothesized that SNHG5 could play a key role in CRC progression by regulating the miR-363-3p/AURKA axis through a ‘competitive endogenous RNA (ceRNA)’ mechanism. This study was performed to investigate the function and potential mechanisms of the SNHG5/miR-363-3p/AURKA regulatory network in CRC progression.

## Material and methods

### Clinical samples

Forty human CRC and corresponding paired adjacent tissues were available from the First Affiliated Hospital of Henan University of Science and Technology. Tissue specimens, after tumor resection, were snap-frozen in liquid nitrogen and cryopreserved at – 196°C. Ethics approval was granted by the Ethics Committee of the First Affiliated Hospital of Henan University of Science and Technology and the Ethics Committee of Xi’an No. 3 Hospital, with informed consent from each enrolled participant prior to the surgery. All procedures followed the principles of the Declaration of Helsinki [30].

### Cell culture and transfection

Cell Resource Center of Shanghai Institutes for Biological Sciences (Shanghai, China) was the provider of CRC cell lines (HCT116, HT-29, SW620, and SW480) and immortalized colonic cells (NCM460). In Dulbecco’s modified Eagle’s medium (DMEM, Hyclone, Logan, UT, USA) containing 10% fetal bovine serum (FBS, Sciencell Research, Carlsbad, CA, USA), the cells were cultured at 37°C in 5% CO_2_.

Shanghai GenePharma Inc. (Shanghai, China) was the provider to synthesize both miR-363-3p mimics or miR-363-3p inhibitors, and negative controls. SNHG5 overexpression plasmids (SNHG5), SNHG5 siRNAs (si-SNHG5), AURKA overexpression plasmids (AURKA), siRNAs targeting AURKA (si-AURKA), and corresponding negative controls were obtained from RiboBio Co., Ltd. (Guangzhou, China). The transfection was conducted employing Lipofectamine™ 3000 (Invitrogen, Carlsbad, CA, USA) [31]. After 24 h, overexpression or inhibition efficiency was detected by qRT-PCR. Preliminary experiments showed that after the transfection, the up-regulation or down-regulation of miR-363-3p expression could at least last for 72 h.

### Quantitative real-time polymerase chain reaction (qRT-PCR)

TRIzol kit (Invitrogen, Thermo Fisher Scientific, Inc., Carlsbad, CA, USA) and PrimeScript™ RT reagent Kit with gDNA Eraser (Takara, Dalian, China) were adopted to extract total RNA and prepare cDNA, respectively. Stem-loop method was adopted to conduct the reverse transcription of miR-363-3p with 5’-CTCAACTGGTGTCGTGGAGTCGGCAATTCAGTTGAGTACAGAUGG-3’ as the sequence of the primer. With a One-Step SYBR® PrimeScript™ PLUS RT-PCR Kit (Takara, Dalian, China), miR-363-3p, AURKA mRNA, and SNHG5 expression levels were quantifying by qRT-PCR, with relative expressions calculated with the 2^−ΔΔCt^ method [32]. Below are the sequences of the primers: miR-363-3p (Forward, 5’-GCCGAGAATTGCACGGTAT-3’; Reverse, 5’-CTCAACTGGTGTCGTGGA-3’), AURKA (Forward, 5’-CAGACTGGATACCGGGACC-3’; Reverse, 5’-CTTCAGCACGTTTTTGCACTG-3’), SNHG5 (Forward, 5’-AAGCTTCTTTTACGTCGGCCTTCGCGAGCGTCTGG-3’; Reverse, 5’-GGATCCTCGAGTTAGTGGATTTTCCATTTAATGCTCC-3’), U6 (Forward, 5’-CTCGCTTCGGCAGCACA-3’; Reverse, 5’-AACGCTTCACGAATTTGCGT-3’), glyceraldehyde-3-phosphate dehydrogenase (GAPDH) (Forward, 5’-ATGTTCGTCATGGGTGTGAA-3’; Reverse, 5’-CAGTGATGGCATGGACTGT-3’), peptidylprolyl isomerase A (PPIA) (Forward, 5’-GGTTCCCAGTTTTTCATTTG-3’; Reverse, 5’-ATGGTGATCTTCTTGCTGGT-3’). U6 was employed to normalize miR-363-3p expression. GAPDH and PPIA worked as the internal references for AURKA and SNHG5, respectively.

### Cell counting kit-8 (CCK-8) assay

CCK-8 assay was conducted as described previously [10]. The transfected SW620 and HT-29 cells were transferred into a 96-well plate (3,000 cells/well). Then, 10 µL of CCK-8 regent (Beyotime, Shanghai, China) was dripped into each well at intervals of 12, 24, 48, 72, and 96 h after cells were seeded. After 2 h, the cell viability was recorded by measuring the absorbance of the cells at 450 nm wavelength with a microplate reader.

### Wound healing assay

Wound healing assay was conducted as described previously [33]. At the time when the cells reached about 80%-90% confluence in culture wells, a pipette tip was utilized to make a scratch on the monolayer cells across the center of the well. Then, the wells were rinsed twice with sterile phosphate buffer saline (PBS), and the detached cells were removed. The scratch was observed with a microscope, and the width of the scratch was recorded. The cells, added with the fresh serum-free medium, were cultured for 1 d. The width of scratch was then observed and recorded again.

### Transwell invasion assay

SW620 and HT-29 cells (5 × 10^4^ cells/well, in serum-free medium) were transferred into the upper compartment of each Transwell chamber (Corning, NY, USA) coated with a layer of Matrigel (Corning, NY, USA), and, meanwhile, 600 μL of medium containing 20% FBS was supplemented in the lower compartment. After 24 h, the invaded cells through the membrane were fixed in paraformaldehyde and then stained with 0.1% crystal violet solution. A microscope (×100) was adopted to count the number of cells [34].

### Flow cytometry

The annexin V-fluorescein isothiocyanate (FITC)/propidium iodide (PI) Apoptosis Detection Kit (Beyotime, Shanghai, China) was used for detecting cell apoptosis. Briefly, 1 × 10^6^ SW620 and HT-29 cells were re-suspended in 1 mL of PBS and centrifuged at 400 × g for 5 min at 4°C. The cells were then re-suspended in 200 µL of PBS and incubated with 10 µL of Annexin V-FITC staining solution and 10 µL of PI staining solution for 30 min at 4°C in the dark. The percentage of apoptotic cells was then analyzed by a flow cytometer (BD Biosciences, San Jose, CA, USA) [35].

### Dual-luciferase reporter assay

Dual-luciferase reporter gene assay was performed based on previous studies [36]. To be specific, reporter vectors (Promega, Madison, WI, USA) carrying the wild-type (WT) or mutant (MUT) SNHG5 sequence or AURKA 3′UTR sequence, together with miR-363-3p mimics or miR-con were co-transfected into SW620 and HT-29 with Lipofectamine™ 3000 (Invitrogen, Carlsbad, CA, USA). After 24 h, the transfected cells were collected to measure the luciferase activities utilizing the dual-luciferase reporter assay kit (Promega, Madison, WI, USA).

### RNA immunoprecipitation (RIP) assay

RIP assay was conducted as described previously [37]. Magna RIP RNA-binding protein immunoprecipitation kit (Millipore, Billerica, MA, USA) was adopted. After the dissolution in complete RIPA buffer containing protease inhibitor cocktail and RNase inhibitor, SW620 and HT-29 cells were incubated with RIP buffer containing magnetic bead conjugated with anti-argonaute 2 (Ago2) (1:50, ab186733, Abcam) or control normal human immunoglobin G (IgG) (1:50, ab181236, Abcam). Then, the immunoprecipitated RNA was isolated by digesting the samples with proteinase K before the co-precipitated RNA was purified. Subsequently, the enrichment of miR-363-3p and AURKA in SW620 and HT-29 cells was quantified by qRT-PCR.

### Immunohistochemistry (IHC) staining

4 mm, formalin-fixed, paraffin-embedded tissue sections were prepared to perform IHC staining. Specifically, the tissue sections were deparaffinized in xylene, rehydrated in ethanol, and immersed in antigenic retrieval buffer for 15 min in a microwave oven, followed by being incubated with 3% hydrogen peroxide for 20 min, and endogenous peroxidase activity was blocked. Then, 5% bovine serum albumin was applied for blocking the unspecific antigens at ambient temperature for 15 min. Subsequently, these sections were incubated with the antibody against AURKA (1:100. Abcam, Shanghai, China) overnight at 4°C and then with secondary antibody for 1 h at room temperature, and the color was developed with 3,3′-diaminobenzidine tetrachloride (DAB). The primary antibody was not utilized in the negative control group. The images were obtained employing an upright microscope (Nikon, Tokyo, Japan). The IHC results were scored by two independent pathologists. The intensity of IHC staining and the proportion of positively stained tumor cells were assessed as described previously [38]. The percentage of stained tumor cells was recorded (0%: 0; <1%: 1; 1–10%: 2; 11–33%:3; 34–66%: 4; >66%: 5), and the degree of staining intensity was also recorded (no staining: 0; weak staining: 1; moderate staining: 2; strong staining: 3). IHC staining score = the percentage of stained tumor cells + staining intensity. The IHC results were grouped according to IHC staining score: negative (IHC staining score: <3), weakly positive (IHC staining score: 4–6), and strongly positive (IHC score: 7–8).

### Western blot

Western blot assay was conducted as described previously [39]. In brief, RIPA lysis solution (Beyotime, Shanghai, China) and a bicinchoninic acid assay kit (Pierce, Rockford, IL, USA) were adopted to conduct total protein extraction and quantification, respectively. Protein samples (20 µg) in each group were loaded for sodium dodecyl sulfate polyacrylamide gel electrophoresis and subsequently transferred onto polyvinylidene fluoride membranes (Millipore, Billerica, MA, USA). After being blocked with 5 defatted milk for 1 h at ambient temperature, the membranes were incubated with primary antibodies against AURKA (ab13824, 1:1000, Abcam), PARP (#9532, 1:1000, Cell Signaling Technology), cleaved caspase-3 (ab32042, 1:1000, Abcam) and GAPDH (ab181602, 1:1000, Abcam) at 4°C overnight, followed by the incubation with horseradish peroxidase coupled secondary antibody at ambient temperature for 1 h. An enhanced chemiluminescence system (Biossci, Wuhan, China) was applied to detect protein signal.

### Statistical analysis

All experiments were conducted three times. The data were processed with GraphPad Prism 8 software (GraphPad Software, San Diego, CA, USA). The comparisons were conducted employing student’s *t*-test or one-way ANOVA. Kaplan-Meier analysis was used for the analysis of survival. Chi-squared test was used for evaluating the correlation between miR-363-3p expression and the clinicopathologic features of CRC patients. *P*< 0.05 indicated statistical significance.

## Results

This study explored the expression pattern and biological function of miR-363-3p in CRC and further investigated its related mechanism. It was revealed that miR-363-3p was lowly expressed in CRC tissues and cells. Overexpression of miR-363-3p inhibited the proliferation, migration, and invasion of CRC cells while inhibition of miR-363-3p had opposite effects. Mechanistically, our study confirmed that AURKA was the target of miR-363-3p in CRC cells. In addition, it was found that high expression of SNHG5 in CRC contributed to the dysfunction of miR-363-3p.

### MiR-363-3p expression was decreased in CRC tissues and cells

**Figure 1.**
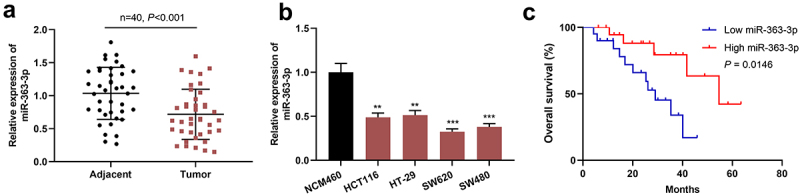
**MiR-363-3p was lowly expressed in CRC tissues and cells** A. qRT-PCR was used to detect the expression of miR-363-3p in 40 CRC tissues (Tumor group) and adjacent normal tissues (Adjacent group), and U6 served as the internal control. B. qRT-PCR was used to detect the expression of miR-363-3p in the immortalized colonic epithelial cell (NCM460) and CRC cells (HCT116, HT-29, SW620, and SW480), and U6 was used as the internal control. C. Kaplan-Meier method was used to compare the survival time of the patients with high or low expression of miR-363-3p. The error bars represented the mean ± SD of at least three independent experiments. Compared with the adjacent group or NCM460 cell, **P< 0.01 and ***P< 0.001.

The data of qRT-PCR indicated that miR-363-3p expression in CRC tissue was markedly decreased (Figure 1(a)). Consistently, miR-363-3p expression was decreased in four CRC cell lines compared with in immortalized colonic epithelial cell line NCM460 (Figure 1(b)). The median of miR-363-3p expression in CRC tissues was used as the cutoff value, and the clinical samples were divided into miR-363-3p high and low expression groups, and the association between miR-363-3p and the pathological parameters of CRC patients was investigated. Chi-square test showed that low miR-363-3p expression in CRC tissues was associated with larger tumor volume and lymph node metastasis (Table 1). In addition, compared with patients with high miR-363-3p expression, CRC patients with low miR-363-3p expression had a lower overall survival rate (Figure 1(c)).

**Table 1.** Relationship between miR-363-3p expression and the clinicopathological characteristics of CRC patients

		miR-363-3p expression			
Characteristics	Number (n = 40)	Low	High	*χ^2^*	*P* value
Sex					
Male	23	12	11	0.1023	0.7491
Female	17	8	9		
Age(years)					
< 60	19	10	9	0.1003	0.7515
≥ 60	21	10	11		
Lymph node metastasis					
Present	24	17	7	10.4167	0.0012
Absent	16	3	13		
Tumor size (cm)					
< 5	12	3	9	4.2857	0.0384
≥ 5	28	17	11		
Histological grade					
Well or moderate	18	7	11	1.6162	0.2036
Poor	22	13	9		
Tumor stage					
T1 – T2	16	7	9	0.4167	0.5186
T3 – T4	24	13	11		
Differentiation					
Well/moderate	19	8	11	0.9023	0.3422
Poor	21	12	9

### MiR-363-3p affected the malignant phenotypes of CRC cells

**Figure 2.**
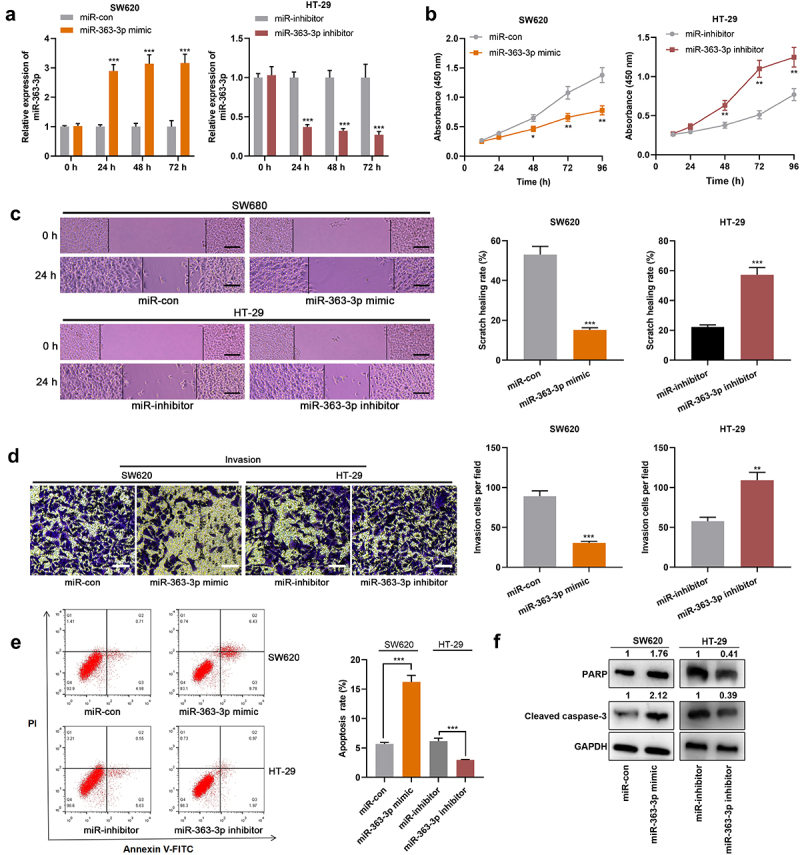
**MiR-363-3p inhibited CRC cell proliferation, migration, and invasion** SW620 cells were transfected with 50 nM of miR-363-3p mimics (or 50 nM of miR-con) while HT-29 cells were transfected with 50 nM of miR-363-3p inhibitor (or 50 nM of miR-inhibitor. A. qRT-PCR was used to detect the relative expression of miR-363-3p in SW620 and HT-29 cells at 24 h, 48 h, or 72 h after transfection, and U6 was used as the internal control. B. CCK-8 experiment was used to detect the proliferation of SW620 and HT-29 cells 24 h after transfection. C. The migration of SW620 and HT-29 cells 24 h after transfection was detected by scratch healing experiment. Scale bar: 100 μm. D. Transwell assay was used to detect the invasion of SW620 and HT-29 cells 24 h after transfection. Scale bar: 100 μm. E. Flow cytometry was used to detect the apoptosis of SW620 and HT-29 cells 24 h after transfection. F. Western blot was used to detect the relative expression level of PARP and cleaved caspase-3 in SW620 and HT-29 cells 24 h after transfection, and GAPDH was used as the internal control. Error bars represented the mean ± SD of at least three independent experiments. Compared with the miR-con or miR-inhibitor group, *P< 0.05, **P< 0.01, and ***P< 0.001.

Among these aforementioned CRC cell lines, SW620 and HT-29 cells exhibited the lowest and highest expression of miR-363-3p, respectively. Therefore, SW620 and HT-29 cells were selected to construct *in vitro* cell models with miR-363-3p overexpression and inhibition, respectively. qRT-PCR showed that the transfection of miR-363-3p mimics significantly increased the expression of miR-363-3p in SW620 cells, and the transfection of miR-363-3p inhibitors significantly reduced the expression of miR-363-3p in HT-29 cells (Figure 2(a)). As shown, miR-363-3p overexpression observably repressed the viability, migration, and invasion of SW620 cells and promoted apoptosis, but the inhibition of miR-363-3p expression facilitated the proliferation, migration, and invasion of HT-29 cells and inhibited apoptosis (Figure 2(b-e)). Furthermore, compared with the control group, the transfection of miR-363-3p mimic increased the expression levels of PARP and cleaved caspase-3 in SW620 cells while downregulating miR-363-3p expression decreased the expression levels of PARP and cleaved caspase-3 in HT-29 cells (Figure 2(f)). These findings indicated that miR-363-3p exhibited an inhibitory effect on the malignant phenotypes of CRC cells.

### AURKA was a direct target of miR-363-3p

**Figure 3.**
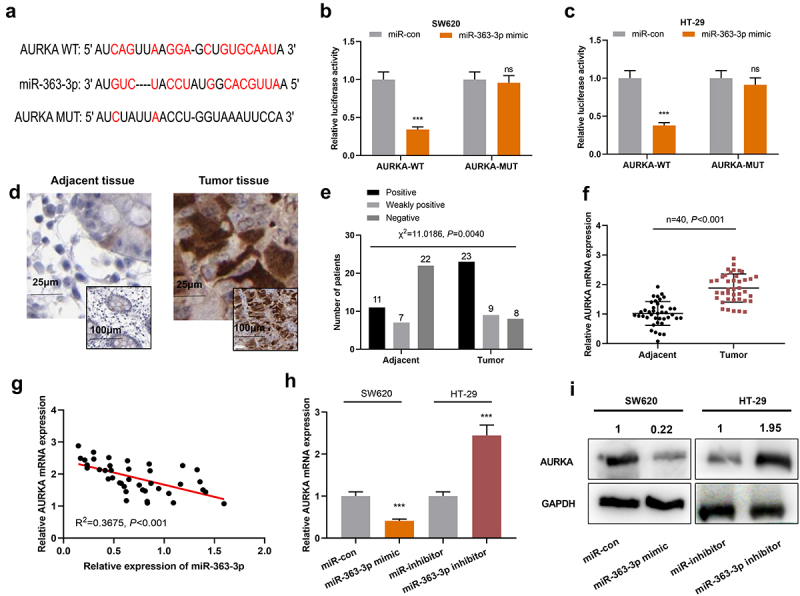
**AURKA was the target of miR-363-3p** A. Bioinformatics analysis (http://starbase.sysu.edu.cn/) was used to predict the binding sequence between miR-363-3p and AURKA 3’-UTR. B-C. Luciferase activities in SW620 and HT-29 cells co-transfected with AURKA-WT or AURKA-MUT and miR-con (50 nM) or miR-363-3p mimic (50 nM) were assessed utilizing dual-luciferase reporter gene experiment. D. IHC was used to detect AURKA expression in 40 cases of CRC tissues (Tumor group) and normal tissues adjacent to cancer (Adjacent group). Chi-square test was used to analyze the positive, weakly positive, and negative staining of AURKA in tumor tissues and adjacent tissues of 40 CRC patients. F. qRT-PCR was used to detect the relative expression of AURKA mRNA in 40 cases of CRC tissues (Tumor group) and adjacent normal tissues (Adjacent group), with GAPDH and PPIA as the internal controls. G. Pearson correlation analysis was used to detect the correlation between miR-363-3p and AURKA mRNA expressions in tumor tissues of 40 CRC patients. H-I. The relative expressions of AURKA mRNA and protein in SW620 cells transfected with 50 nM of miR-363-3p mimic (or 50 nM of miR-con) and HT-29 cells transfected with 50 nM of miR-363-3p inhibitor (or 50 nM of miR-inhibitor) were detected by qRT-PCR and Western blot. Error bars represented the mean ± SD of at least three independent experiments. Compared with the miR-con or miR-inhibitor group, *P< 0.05, ***P< 0.001, and ns: *P*> 0.05.

For deciphering how miR-363-3p regulated CRC progression, possible target genes of miR-363-3p were screened with StarBase database, the findings of which suggested that AURKA was one of the candidate targets of miR-363-3p (Figure 3(a)). Interestingly, the high expression of AURKA was associated with lymph node metastasis and larger tumor size in the enrolled CRC patients (Table 2). As shown, miR-363-3p overexpression repressed the luciferase activity of AURKA-WT; after the predicted binding site was mutated, miR-363-3p could not reduce the luciferase activity of the reporter vector (Figure 3(a-c)). Furthermore, the positive staining rate of AURKA was higher in CRC tissues compared with adjacent tissues, indicating that AURKA expression was increased in CRC tissues than in normal colonic tissues; additionally, the nuclear staining of AURKA was stronger in tumor tissues than normal tissues (Figure 3(d-e)). Meanwhile, qRT-PCR showed that AURKA mRNA was remarkably highly expressed in CRC tissues (Figure 3(f)). Moreover, miR-363-3p expression was negatively correlated with AURKA mRNA expression in CRC tissues (Figure 3(g)). Besides, the transfection of miR-363-3p mimics suppressed AURKA expression in SW620 cells whereas the transfection of miR-363-3p inhibitors promoted AURKA expression in HT-29 cells (Figure 3(h-i)). Altogether, a conclusion was drawn that AURKA was a direct target of miR-363-3p in CRC cells.

**Table 2.** Relationship between AURKA expression and the clinicopathological characteristics of CRC patients

		AURKA expression			
Characteristics	Number (n = 40)	Low	High	*χ^2^*	*P* value
Sex					
Male	23	10	13	0.9207	0.3373
Female	17	10	7		
Age (years)					
< 60	19	9	10	0.1003	0.7515
≥ 60	21	11	10		
Lymph node metastasis					
Present	24	8	16	6.6667	0.0098
Absent	16	12	4		
Tumor size (cm)					
< 5	12	10	2	7.6190	0.0058
≥ 5	28	10	18		
Histological grade					
Well or moderate	18	11	7	1.6162	0.2036
Poor	22	9	13		
Tumor stage					
T1 – T2	16	7	9	0.4167	0.5186
T3 – T4	24	13	11		
Differentiation					
Well/moderate	19	12	7	2.5063	0.1134
Poor	21	8	13

### AURKA counteracted the effects of miR-363-3p on the malignant phenotypes of CRC cells

**Figure 4.**
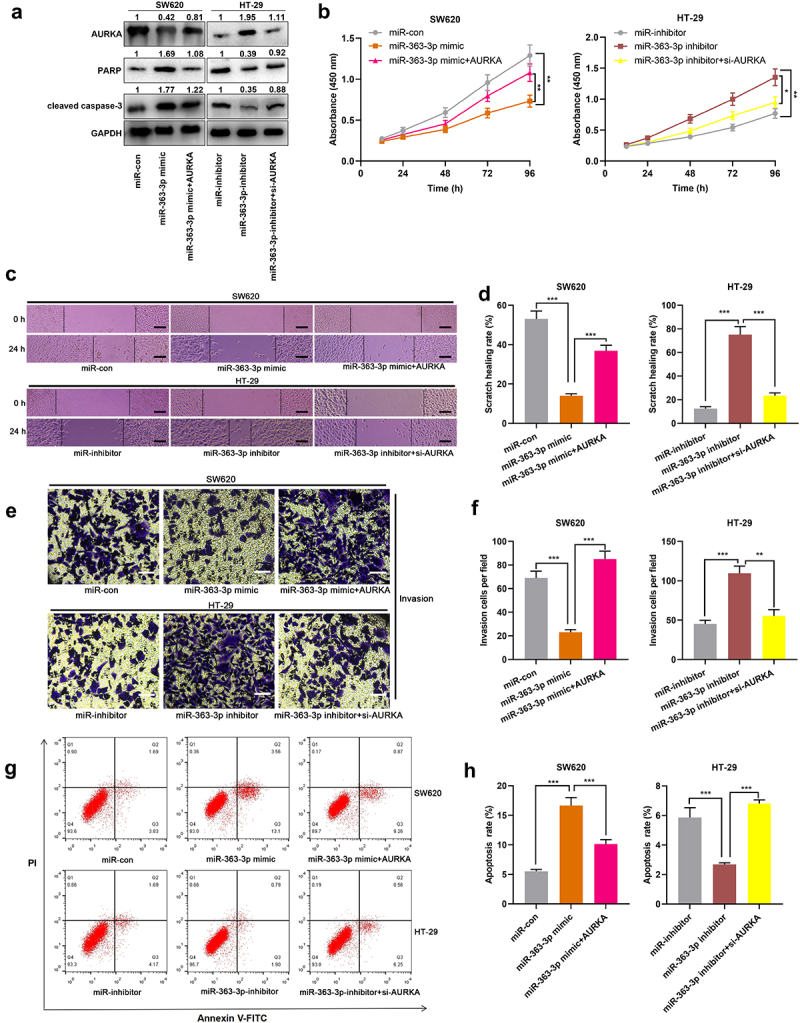
**MiR-363-3p/AURKA axis participated in regulating the proliferation, migration, and invasion of CRC cells** SW620 cells were transfected with 50 nM of miR-363-3p mimics or co-transfected with 50 nM of AURKA overexpression plasmid while HT-29 cells were transfected with 50 nM of miR-363-3p inhibitor or co-transfected with 50 nM of AURKA siRNA (si-AURKA). A. Western blot was used to detect the relative expression of AURKA, PARP, and cleaved caspase-3 protein in SW620 and HT-29 cells after transfection or co-transfection, and GAPDH was used as internal control. B. The proliferation of SW620 and HT-29 cells after transfection or co-transfection was detected utilizing CCK-8 method. C-D. The migration of SW620 and HT-29 cells after transfection or co-transfection was detected employing scratch healing experiment. Scale bar: 100 μm. E-F. Transwell experiment was used to detect the invasion of SW620 and HT-29 cells after transfection or co-transfection. Scale bar: 100 μm. G-H. Flow cytometry was used to detect the apoptosis of SW620 and HT-29 cells after transfection or co-transfection. Error bars represented the mean ± SD of at least three independent experiments. Compared with the miR-con, miR-inhibitor, miR-363-3p mimic, or miR-363-3p inhibitor group, *P< 0.05, **P< 0.01, and ***P< 0.001.

Next, miR-363-3p mimics and AURKA overexpression plasmid were co-transfected into SW620 cells, while AURKA siRNA was transfected into HT-29 cells together with miR-363-3p inhibitors, and Western blot was adopted to detect the efficiency of co-transfection. The results showed that compared with miR-363-3p mimics group, the co-transfection of miR-363-3p mimics and AURKA overexpression plasmid increased the expression of AURKA and decreased the expressions of PARP and cleaved caspase-3 in SW620 cells; compared with miR-363-3p inhibitors group, in miR-363-3p inhibitor+si-AURKA group, the expression of AURKA in HT-29 cells was decreased, and the expressions of PARP and cleaved caspase-3 were increased (Figure 4(a)). The suppression of proliferation, migration, and invasion of SW620 cells, induced by miR-363-3p overexpression, was partially reversed by AURKA overexpression; on the other hand, knockdown of AURKA counteracted the promoting effects of miR-363-3p inhibitors on the malignant phenotypes of HT-29 cells (Figure 4(b-f)). In addition, overexpression of AURKA attenuated the promoting effects of miR-363-3p overexpression on SW620 cell apoptosis; AURKA knockdown reversed the inhibitory effects of miR-363-3p inhibitor on HT-29 cell apoptosis (Figure 4(g-h)). These phenomena suggested the tumor-suppressive functions of miR-363-3p in CRC were partly dependent on its regulatory function on AURKA.

### SNHG5 regulated miR-363-3p expression by serving as a ceRNA

**Figure 5.**
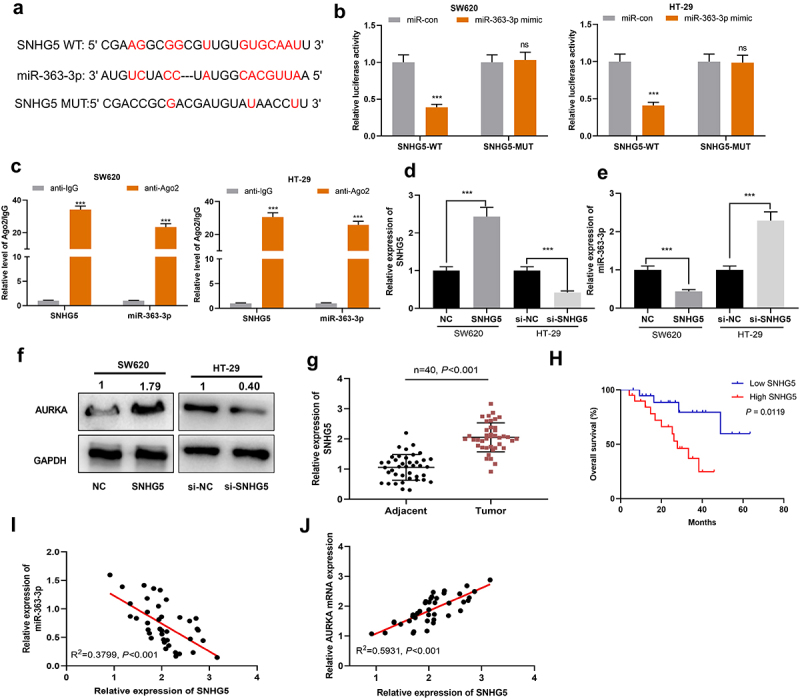
**SNHG5 targeted miR-363-3p in CRC cells** A. Bioinformatics analysis (http://starbase.sysu.edu.cn/) was used to predict the binding sequence between miR-363-3p and SNHG5. B. Luciferase activities were detected in SW620 and HT-29 cells co-transfected with SNHG5-WT or SNHG5-MUT and 50 nM of miR-con or 50 nM of miR-363-3p mimics utilizing dual-luciferase reporter gene experiments. C. RIP assay showed the binding relationship between miR-363-3p and SNHG5 in the anti-Ago2 group of SW620 and HT-29 cells, and anti-IgG was used as the negative control. D. The relative expression of SNHG5 in SW620 and HT-29 cells transfected with 50 nM of SNHG5 overexpression plasmid or 50 nM of si-SNHG5 was detected by qRT-PCR. GAPDH and PPIA were used as the internal controls. E. The relative expression of miR-363-3p in SW620 and HT-29 cells transfected with 50 nM of SNHG5 overexpression plasmid or 50 nM of si-SNHG5 was detected by qRT-PCR. U6 was used as the internal control. F. Western blot was used to detect the expression of AURKA protein in SW620 and HT-29 cells transfected with 50 nM of SNHG5 overexpression plasmid or 50 nM of si-SNHG5, and GAPDH was used as internal control. G. qRT-PCR was used to detect the relative expression of SNHG5 in 40 cases of CRC tissues (Tumor group) and normal tissues adjacent to cancer (Adjacent group), with GAPDH and PPIA as the internal controls. H. Kaplan-Meier method was used to compare the survival time of the patients with high or low expression of SNHG5. I. The correlation between SNHG5 expression and miR-363-3p expression in 40 cases of CRC tissues was analyzed by Pearson correlation analysis. J. The correlation between SNHG5 and AURKA mRNA expressions in 40 cases of CRC tissues was analyzed by Pearson correlation analysis. Error bars represented the mean ± SD of at least three independent experiments. Compared with the miR-con, anti-IgG, and NC or si-NC group, ***P< 0.001 and ns:*P*> 0.05.

LncRNAs are reported to function as ceRNAs, which sponge miRNAs to reduce their availability, thereupon regulating the biological processes of cancer cells [26,27]. Herein, StarBase database was searched for screening the lncRNAs potentially targeting miR-363-3p, and it was found that SNHG5 sequence contained a potential binding site of miR-363-3p (Figure 5(a)). Notably, high expression of SNHG5 was associated with lymph node metastasis in the enrolled CRC patients (Table 3). Subsequently, dual-luciferase reporter experiment was used for validating this prediction, the result of which showed that miR-363-3p mimics repressed the luciferase activity of SNHG5-WT whereas miR-363-3p had no marked effects on that of SNHG5-MUT (Figure 5(b)). Moreover, RIP assay showed that miR-363-3p and SNHG5 were enriched in the anti-Ago2 group as opposed to the anti-IgG group (Figure 5(c)). Next, SNHG5 overexpression plasmid and SNHG5 siRNA were transfected into SW620 cells and HT-29 cells, respectively (Figure 5(d)). In SW620 cells with SNHG5 overexpression, miR-363-3p and AURKA expressions were down-regulated and up-regulated, respectively; in HT-29 cells with SNHG5 knockdown, the opposite results were observed (Figure 5(e-f)). Furthermore, SNHG5 was observed to be overexpressed in CRC tissues (Figure 5(g)). In addition, the overall survival rate of CRC patients with high SNHG5 expression was shorter than that of patients with low SNHG5 expression (Figure 5(h)). Notably, in CRC tissues, SNHG5 expression was negatively correlated with miR-363-3p expression but positively associated with AURKA mRNA expression (Figure 5(i-j)). On all accounts, these findings substantiated that SNHG5 was capable of directly targeting miR-363-3p and positively regulating AURKA expression.

**Table 3.** Relationship between lncRNA SNHG5 expression and the clinicopathological characteristics of CRC patients

		lncRNA SNHG5 expression			
Characteristics	Number (n = 40)	Low	High	*χ^2^*	*P* value
Sex					
Male	23	9	14	2.5575	0.1098
Female	17	11	6		
Age(years)					
< 60	19	12	7	2.5063	0.1134
≥ 60	21	8	13		
Lymph node metastasis					
Present	24	8	16	6.6667	0.0098
Absent	16	12	4		
Tumor size (cm)					
< 5	12	7	5	0.4762	0.4902
≥ 5	28	13	15		
Histological grade					
Well or moderate	18	10	8	0.4040	0.5250
Poor	22	10	12		
Tumor stage					
T1 – T2	16	9	7	0.4167	0.5186
T3 – T4	24	11	13		
Differentiation					
Well/moderate	19	12	7	2.5063	0.1134
Poor	21	8	13

### SNHG5/miR-363-3p/AURKA axis was involved in regulating the biological process of CRC cells

**Figure 6.**
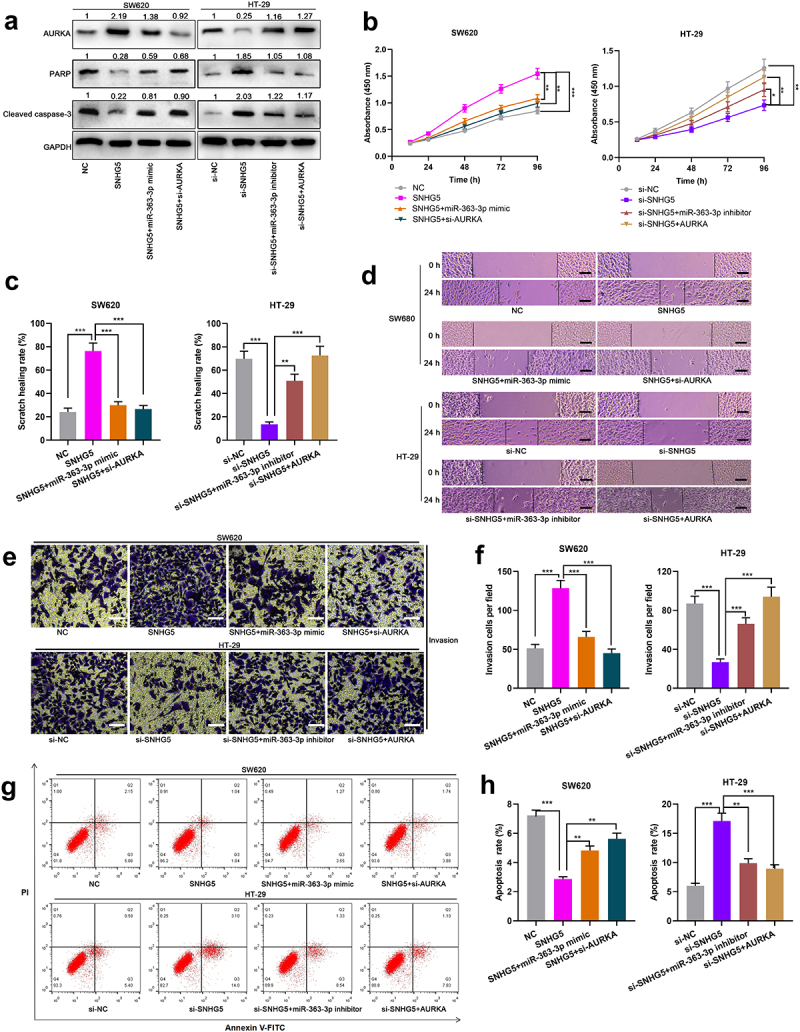
**SNHG5 promoted the proliferation, migration, and invasion of CRC cells by targeting miR-363-3p/AURKA axis** SW620 cells were transfected with the SNHG5 overexpression plasmid (SNHG5, 50 nM) or co-transfected with miR-363-3p mimic (50 nM) or si-AURKA (50 nM) while HT-29 cells were transfected with si-SNHG5 (50 nM) or co-transfected with miR-363-3p inhibitor (50 nM) or AURKA overexpression plasmid (AURKA, 50 nM). A. The relative expressions of AURKA, PARP, and cleaved caspase-3 protein in SW620 and HT-29 cells after transfection or co-transfection were detected employing Western blot, and GAPDH was used as internal control. B. The proliferation of SW620 and HT-29 cells after transfection or co-transfection was detected utilizing CCK-8 method. C-D. The migration of SW620 and HT-29 cells after transfection or co-transfection was detected by scratch healing experiment. Scale bar: 100 μm. E-F. Transwell experiment was used to detect the invasion of SW620 and HT-29 cells after transfection or co-transfection. Scale bar: 100 μm. G-H. Flow cytometry was used to detect the apoptosis of SW620 and HT-29 cells after transfection or co-transfection. Error bars represented the mean ± SD of at least three independent experiments. Compared with the NC, si-NC, SNHG5, or si-SNHG5 group, *P< 0.05, **P< 0.01, and ***P< 0.001.

Subsequently, SW620 cells were co-transfected with SNHG5 overexpression plasmid and miR-363-3p mimics or si-AURKA, and HT-29 cells were co-transfected with SNHG5 siRNA and miR-363-3p inhibitors or AURKA overexpression plasmid (Figure 6(a)). Western blot showed that overexpression of SNHG5 increased the expression of AURKA and decreased the expressions of PARP and cleaved caspase-3 in SW620 cells, while the transfection of miR-363-3p mimics or si-AURKA attenuated this effect; the expression of AURKA in HT-29 cells with SNHG5 knockdown was decreased, and the expressions of PARP and cleaved caspase-3 were increased, while downregulating miR-363-3p expression or overexpression of AURKA reversed this effect (Figure 6(a)). It was also revealed that, SNHG5 overexpression accelerated the proliferation, migration, and invasion of CRC cells and inhibited apoptosis, whereas these effects were counteracted by the co-transfection of miR-363-3p mimics or si-AURKA (Figure 6(b-f)); SNHG5 knockdown suppressed the proliferation, migration, and invasion of CRC cells and promoted apoptosis, whereas these effects were offset after the co-transfection of miR-363-3p inhibitors or AURKA overexpression plasmid (Figure 6(b-f)). Therefore, it was concluded that SNHG5 could promote the malignant phenotypes of CRC cells by targeting miR-363-3p and up-regulating AURKA expression.

## Discussion

A series of miRNAs are dysregulated in human malignancies [8,9]. MiRNAs are considered to be essential regulators in tumorigenesis and cancer progression. For instance, miR-875-3p, whose expression is down-regulated in CRC, represses CRC development by targeting PLK1 [40]. MiR-143-3p blocks CRC cell metastasis by repressing ITGA6 and ASAP3 expressions [41]. Reportedly, miR-363-3p expression is decreased in multiple cancers. Specifically, miR-363-3p is capable of suppressing the proliferation and invasion of osteosarcoma cells by targeting SOX4 [10]. Decreased miR-363-3p expression contributes to the drug resistance of non-small cell lung cancer cells by regulating CUL4A expression [11]. Importantly, a previous study reports that, miR-363-3p, which impedes CRC cell growth and metastasis by targeting SphK2, is lowly expressed in CRC, and its under-expression is linked to the unfavorable prognosis of the patients [12]. Consistent with the findings mentioned above, the current research validated that miR-363-3p was lowly expressed in CRC tissues and cells, which was interrelated to larger tumor size and lymph node metastasis of the patients. *In vitro* assays showed that miR-363-3p overexpression remarkably suppressed the malignancy of CRC cells whereas miR-363-3p inhibition exhibited opposite functions. These data showed that miR-363-3p was a tumor suppressor in CRC.

AURKA, a serine/threonine kinase, regulates centrosome separation, maturation, and spindle assembly and stability [13,42,43]. Its inhibition triggers cell cycle arrest in G2/M phase and apoptosis [44]. Abnormally expressed AURKA is linked to cancer biology in recent years. AURKA overexpression facilitates the migration and invasion of lung cancer cells and head and neck squamous cancer cells by activating Akt/FAK pathway [14,15]. In breast cancer, excessive AURKA expression is related to drug resistance and the detrimental prognosis of patients [45]. Additionally, AURKA expression is regulated by a series of miRNAs, miR-124-3p [46], miR-490-3p [47], and miR-4715-3p [48] included. MiR-363-3p, herein, was revealed to be capable of targeting AURKA and negatively regulating its expression in CRC cells.

LncRNAs are important regulators in many physiological and pathological processes [20,24–26]. Specifically, lncRNA BDNF-AS reduces the viability and migration of CRC cells by epigenetically inhibiting GSK-3β expression [49]. As an oncogenic lncRNA, SNHG6 promotes the malignant biological behaviors of CRC cells by targeting UPF1 to activate TGF-β/Smad signaling pathway as well as up-regulate ZEB1 expression [50]. SNHG5, abnormally expressed in a variety of tumors, has a close relationship with carcinogenesis, cancer progression, and patients’ prognosis. In bladder cancer, SNHG5 overexpression promotes cancer cell proliferation by targeting p27 [25]. In glioma, SNHG5 is highly expressed, and its knockdown blocks the viability and invasion of glioma cells through regulating Wnt/β-catenin signal pathway [51]. These findings theoretically support that SNHG5 can probably be a biomarker and therapy target for cancer diagnosis and treatment. Additionally, lncRNA is able to function as ceRNA and interact with miRNA. For instance, SNHG5 facilitates the growth and invasion of melanoma cells by targeting miR-26a-5p and elevating TRPC3 expression [52]. In this study, SNHG5 was observed to decoy miR-363-3p and negatively modulate its expression. Furthermore, we demonstrated that SNHG5, whose expression was positively correlated with AURKA expression in CRC tissues, could positively regulate AURKA expression in CRC cells through sponging miR-363-3p. Notably, SNHG5 overexpression facilitated the proliferation, migration, and invasion of CRC cells, and inhibits cell apoptosis, and these effects were eliminated by miR-363-3p mimics or AURKA knockdown. These data, to some extent, clarified the upstream regulatory mechanism of miR-363-3p dysregulation in CRC, and showed the ceRNA network composed of SNHG5, miR-363-3p, and AURKA was involved in CRC progression.

## Conclusion

**Figure 7.**
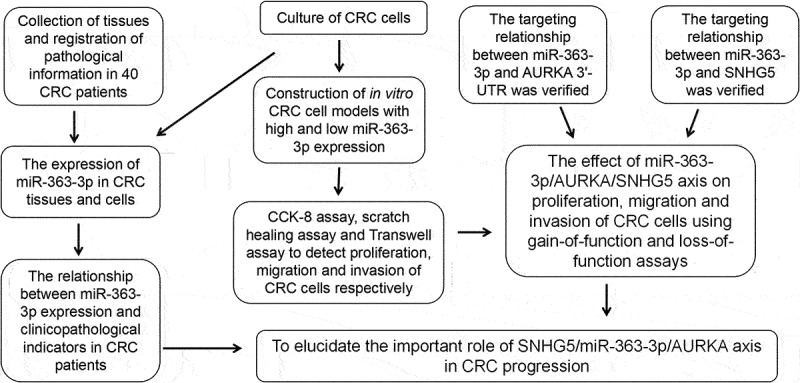
Flow chart of the present study.

All in all, though a series of experiments (Figure 7), we demonstrate miR-363-3p is lowly expressed in CRC and exhibits an anti-cancer role in CRC progression. It targets AURKA to suppress the malignancy of CRC cells, and the down-regulation of its expression is partly due to the overexpression of SNHG5. Our findings deepen the understanding of the mechanism of CRC tumorigenesis and development.

## Data Availability

All data generated or analyzed during this study are available from the corresponding author.

## Abbreviations

miR-363-3p: microRNA-363-3p; SNHG5: small nucleolar RNA host gene 5; AURKA: aurora kinase A; CRC: colorectal cancer; miRNA: microRNA; lncRNA: long noncoding RNA; ceRNA: competitively endogenous RNA; DMEM: Dulbecco’s modified Eagle’s medium; FBS: fetal bovine serum; CCK-8: cell counting kit-8; qRT-PCR: quantitative reverse transcriptase polymerase chain reaction; WT: wild-type; MUT: mutant; IHC: immunohistochemistry; PPIA: peptidylprolyl isomerase A.

## Highlights

1. MiR-363-3p expression is down-regulated in CRC tissues and cells.

2. MiR-363-3p inhibits the viability, migration, and invasion of CRC cells, and promotes cell apoptosis.

3. MiR-363-3p directly targets AURKA and negatively regulates the expression of AURKA in CRC cells.

4. SNHG5 promotes CRC progression by upregulating AURKA expression through sponging miR-363-3p.
